# Effect of Gastrointestinal Digestion on the Bioaccessibility of Phenolic Compounds and Antioxidant Activity of Fermented *Aloe vera* Juices

**DOI:** 10.3390/antiox11122479

**Published:** 2022-12-16

**Authors:** Ruth B. Cuvas-Limon, Pedro Ferreira-Santos, Mario Cruz, José A. Teixeira, Ruth Belmares, Clarisse Nobre

**Affiliations:** 1 Food Research Department, School of Chemical Sciences, Autonomous University of Coahuila, Boulevard Venustiano Carranza e Ing. José Cárdenas s/n Col. República C.P., Saltillo 25280, Coahuila, Mexico; 2 Centre of Biological Engineering, University of Minho, Campus de Gualtar, 4710-057 Braga, Portugal; 3 LABBELS—Associate Laboratory, 4710-057 Braga, Portugal; 4 Department of Food Science and Technology, Antonio Narro Autonomous Agricultural University, Calzada Antonio Narro, No. 1923 Col. Buena Vista C.P., Saltillo 25315, Coahuila, Mexico

**Keywords:** plant-based beverages, fermented beverages, probiotics, natural bioactive compounds, in vitro digestion, biotransformation of phenolic compounds

## Abstract

Plant-based beverages are enriched by the fermentation process. However, their biocompounds are transformed during gastrointestinal digestion, improving their bioaccessibility, which is of primary importance when considering the associated health benefits. This study aimed to evaluate the effect of in vitro gastrointestinal digestion on phenolic compound bioaccessibility and antioxidant activity of novel *Aloe vera* juices fermented by probiotic *Enterococcus faecium* and *Lactococcus lactis*. *Aloe vera* juices were digested using the standardized static INFOGEST protocol. During digestion, phenolic compounds and antioxidant activity (DPPH, ABTS, and FRAP) were accessed. The digestion process was seen to significantly increase the total phenolic content of the fermented *Aloe vera* juices. The fermentation of *Aloe vera* increased the bioaccessibility of juice biocompounds, particularly for kaempferol, ellagic acid, resveratrol, hesperidin, ferulic acid, and aloin. The phenolics released during digestion were able to reduce the oxidative radicals assessed by ABTS and FRAP tests, increasing the antioxidant action in the intestine, where they are absorbed. The fermentation of *Aloe vera* by probiotics is an excellent process to increase the bioavailability of beverages, resulting in natural added-value functional products.

## 1. Introduction

The food industry has been seeking to innovate its products in order to provide foods that are not only nutritious but also have added functionality, leading to improved human health and well-being and/or reduced risk of disease [1,2]. In this context, plant-based beverages, made with either fruits or vegetables fermented with probiotics, appear to be an important category of functional foods. Plant-based beverages are excellent sources of vitamins, minerals, and bioactives. At the same time, they represent a good option when choosing a drink containing probiotics, which are usually dairy-based matrices, due to their hydration properties, freshness, and attractive flavors, in addition to being lactose-free, milk protein-free, and strictly vegetarian [3].

The fermentation process itself increases the release of the plant’s bioactive compounds, increases bioaccessibility and bioavailability, and consequently improves the nutritional and functional properties of the food, with beneficial effects on health. Beverages fermented with probiotic bacteria may act as vehicles for the delivery of probiotic species into the gut, having a fundamental role in microbiota modulation [1,4]. Even pasteurized fermented juices with probiotic bacteria such as Lactobacilli may have similarly beneficial health effects as described for probiotics due to the presence of Lactobacilli-secreted products generated during the fermentation [5].

Lactic acid bacteria (LAB) can (bio)transform polyphenols during the fermentation process into simple phenolic compounds with improved bioactivity. These compounds are bio-transformed again during gastrointestinal digestion, and their content and bioactivity (e.g., antioxidant) can be changed [3]. Therefore, simulation of in vitro gastrointestinal digestion of food has been widely used to evaluate bioavailability and to study structural changes, release efficiency, digestibility, stability, and bioaccessibility of food nutrients [6,7,8,9], even if phenolic biotransformation by the gut microbiota is not always performed prior to tests of bioaccessibility and bioavailability.

The most frequently found LAB species in commercialized probiotic products include *Lactobacillus*, *Bifidobacterium*, *Lactococcus*, and *Enterococcus* [10]. *Lactococcus lactis* and *Enterococcus faecium* have been referred to as some of the strains most widely used in the food industry [10]. *E. faecium* has been isolated from human milk [10,11]. *E. faecium* has been used in the production of different fermented food products, such as artisanal cheese, sausages, and olives, mainly because it plays an important role in the development of organoleptic characteristics due to proteolytic and lipolytic activity [11]. Although few studies have demonstrated their probiotic potential, they have been shown to possess several health properties similar to probiotics, such as immune-modulatory activity and anti-inflammatory properties, as well as the ability to produce antimicrobial compounds against pathogens, to lower cholesterol, and to adhere to intestinal cells [11,12,13]. On the other hand, *L. lactis* strains have been recognized as probiotics [10]. They have been shown to improve both gut immunomodulation and fasting insulin, and to control cholesterol and hypertension [14,15]. Having GRAS status (Generally Recognized as Safe), they have been used as commercial starter cultures in vegetables and in the dairy industry to ferment milk into buttermilk, cheese, yoghurt, and sour cream [15].

A wide variety of research on fermented beverage production may be found in the literature, including fruits such as oranges, apples, lemons, passion fruits, pomegranates, carrots, etc. [16], and more recently *Aloe vera* [17,18]. *Aloe vera* contains polysaccharides, proteins and amino acids, minerals, phenolics, anthraquinones, and enzymes in its composition [19]. Due to the chemical and functional properties of these compounds, *Aloe vera* has been largely used for wounds and inflammation and has been used in the treatment and prevention of several diseases, namely cancer, ulcers, diabetes, microbial and skin diseases, and others [20]. Therefore, the production of juices based on *Aloe vera* may result in very interesting products for consumers and ultimately for the food industry market.

To our knowledge, there is no report on the release efficiency and bioavailability of phytochemicals from the fermentation of *Aloe vera* juice inoculated with *E. faecium* and *L. lactis* during in vitro digestion. Therefore, this study aims to explore the behavior of bioactive compounds, antioxidant activity, and bioaccessibility of *Aloe vera* juice fermented with LAB during simulated in vitro gastrointestinal digestion. It may provide useful information to promote the development of functional beverages with added value, promoting the use of natural resources that improve human health.

## 2. Materials and Methods

### 2.1. Materials and Reagents

Pepsin, pancreatin, Folin–Ciocâlteu phenol reagent, 2,2-diphenyl-1-picrylhydrazyl (DPPH), 2,2′–azinobis-(3-ethylbenzothiazoline-6-sulfonate) (ABTS), 2,4,6-tris(2-pyridyl)-S-triazine (TPTZ), gallic acid, 6-hydroxy-2,5,7,8-tetramethylchroman-2-carboxylic acid (Trolox), vanillic acid, chlorogenic acid, catechin, epicatechin, *p*-coumaric acid, ellagic acid, naringenin, hesperidin, resveratrol, ferulic acid, quercetin, 3,4-dihydroxybenzoic, taxifolin, aloin, kaempferol, ferric chloride hexahydrate, sodium acetate trihydrate, glacial acetic acid, hydrochloric acid, and sodium acetate trihydrate were purchased from Sigma-Aldrich Ltd. (St. Louis, MO, USA). All chemicals used were of analytical grade, and the water was ultra-pure Milli-Q water.

### 2.2. Juice Preparation and Fermentation

Fresh whole *Aloe barbadensis* Miller (*Aloe vera*) leaves (four years old) were purchased from Ulíavera (Córdoba, Spain), and harvested from Ibiza plantations. Leaves were washed and disinfected (2% sodium hypochlorite solution) and rinsed with distilled water. The spikes and the inferior and superior parts of each leaf were removed before longitudinally slicing of the leaf to separate the epidermis from the parenchyma (gel). The gel was pressed using a laboratory manual roll processor, lyophilized, and stored at −20 °C until further use.

The lyophilized gel of *Aloe vera* (2 g) was subject to an enzymatic-assisted solid–liquid extraction with cellulase (1.5 mg/mL) in a water volume of 60 mL. The reaction occurred for 1 h at 45 °C in a 200 mL cylindrical reactor, duly protected from light, in a thermostatic water bath with agitation (150 rpm). The supernatants were then filtered through Whatman No. 4 filter paper. Finally, the *Aloe vera* samples were sterilized with a 0.22 µm membrane. Juice conditions applied were previously studied in another work [18].

For *Aloe vera* juice fermentation, two LABs were used: *Enterococcus faecium* (isolated from human breast milk) and *Lactococcus lactis* BS-10 (Chr Hansen, Hørsholm, Denmark). Fermentation was carried out using the same conditions as reported in previous work [18]. Briefly, 100 mL of *Aloe vera* was inoculated with 1400 µL of each LAB (1 × 10^6^ CFU/mL) for 48 h at 37 °C. After fermentation, samples were collected and stored at −20 °C until in vitro digestion analysis. The experiments were performed in triplicate.

### 2.3. Procedure for In Vitro Digestion

The harmonized INFOGEST in vitro digestion model (static method) mimicking the mouth, stomach, and small intestine conditions [21] was applied to the digestion of the *Aloe vera* juices (non-fermented and fermented ones), with small modifications [22,23]. Table 1 shows the composition of the electrolyte stock solutions used during in vitro digestion, namely, simulated salivary fluid (SSF), simulated gastric fluid (SGF), and simulated intestinal fluid (SIF).

In the oral phase, 5 mL of samples (previously kept in a water bath for the necessary time until reaching 37 °C under constant agitation (120 rpm)) were exposed to simulated mouth conditions (SSF solution and CaCl_2_ (H_2_O)_2_). Samples were returned to the shaking water bath and incubated at 37 °C for 2 min. Note that α-amylase was not used since the samples did not contain starch.

For the stomach simulation, porcine pepsin solution (with a final activity of 2000 U/mL), SGF solution, and CaCl_2_ (H_2_O)_2_ (0.15 mmol/L) were added. The pH was adjusted to 3 (HCl, 1 mol/L) and the volume was completed with Milli-Q water. Samples were incubated at 37 °C for 2 h. After the gastric phase, a sample was taken and cooled in ice to stop the enzymatic reaction.

Finally, for the intestinal phase simulation, SIF solution, CaCl_2_ (H_2_O)_2_ (0.6 mmol/L), pancreatin (with a final activity of 100 U/mL), and bile solutions (10 mmol/L in the final volume) were added to the gastric phase. The pH was adjusted to 7 (NaOH, 1 mol/L), and the final volume was adjusted with Milli-Q water. The mixture was incubated for 2 h at 37 °C. At the end of the intestinal phase, the reaction was stopped by adding 1 mmol/L of an enzyme inhibitor (Pefabloc SC). Samples were digested at least in triplicate.

**Figure 1:**
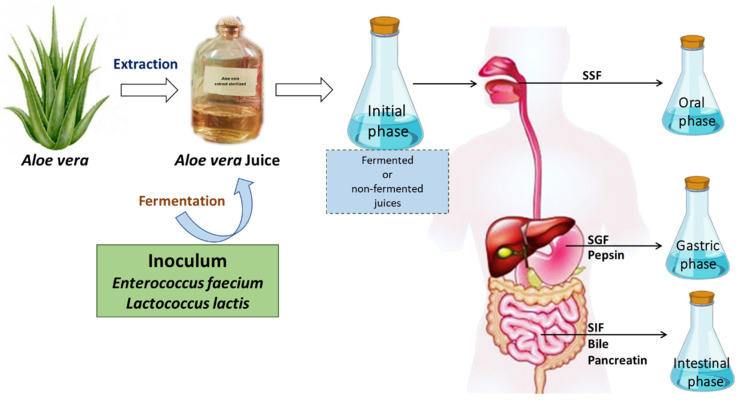
Juice preparation procedure and in vitro gastrointestinal digestion (static model) of non-fermented *Aloe vera* and *Aloe vera* fermented either with *Enterococcus faecium* or with *Lactococcus lactis*.

A representative diagram of the preparation of *Aloe vera* juices and the gastrointestinal digestion system is shown in Figure 1.

### 2.4. Total Phenolic Content Analysis

The total phenolic content (TPC) was determined using a 96-well microplate colorimetric assay by the Folin–Ciocâlteu method adapted from Ferreira-Santos et al. [24]. Briefly, 10 µL of the sample was mixed with 60 µL of Na_2_CO_3_ (75 g/L), 15 µL of Folin–Ciocâlteu reagent, and 200 µL of Milli-Q water and incubated at 60 °C for 5 min. The absorbance was measured at 700 nm using a spectrophotometer (Synergy HT, Biotek Instruments, Inc., Winooski, VT, USA). A standard curve was performed using gallic acid (1500–50 mg/L, R^2^ = 0.99), and the results were expressed in mg of gallic acid equivalents (GAE) per liter of *Aloe vera* juice (mg/L).

### 2.5. Identification of Individual Phenolic Compounds by Ultra-Performance Liquid Chromatography (UPLC-DAD)

Individual phenolic compounds were identified and quantified by ultra-performance liquid chromatography (UPLC) as defined and validated by Ferreira-Santos et al. [24]. A Shimadzu Nexpera X2 UPLC chromatograph equipped with a diode array detector (DAD) (Shimadzu, SPD-M20A, Columbia, MD, USA) was used. Separation was performed at 40 °C on a reversed-phase Aquity UPLC BEH C18 column (2.1 mm × 100 mm, 1.7 µm) from Waters (Milford, MA, USA) and eluted with water/formic acid (0.1%) and 100% acetonitrile at a flow rate of 0.4 mL/min. The biocompounds were identified and quantified by comparison with their UV spectra (wavelengths 209–370 nm) and retention times with that of corresponding standards. Calibration curves were performed for each compound at concentrations between 250–2.5 mg/L (vanillic acid, chlorogenic acid, catechin, epicatechin, *p*-coumaric acid, ellagic acid, naringenin, hesperidin, resveratrol, ferulic acid, quercetin, 3,4-dihydroxybenzoic, taxifolin, aloin, and kaempferol (R^2^ > 0.99)). Results were expressed in milligrams per liter (mg/L).

### 2.6. Antioxidant Activity

#### 2.6.1. Ferric Reducing Antioxidant Power Assay (FRAP)

A FRAP test was performed according to the method described by Benzie and Strain [25], with a few modifications. A volume of 10 µL of sample (properly diluted and filtered) was mixed with 290 µL of FRAP reagent (Tripyridil-s-triazine, FeCl_3_, and acetate buffer). The resultant reaction mixture was incubated at 37 °C for 15 min. The absorbance was measured at 593 nm in a spectrophotometric microplate reader (Synergy HT, Biotek Instruments, Inc., USA), against water as a blank. A standard curve was prepared using an aqueous solution of FeSO_4_ 7H_2_O (200 to 1000 µM). FRAP values were expressed as micromoles of ferrous equivalent per liter of *Aloe vera* juice (µmol Fe (II)/L).

#### 2.6.2. ABTS Radical Cation Scavenging Activity

The radical cation decolorization (ABTS) assay was performed according to the method described by Ferreira-Santos et al. [12], with minor modifications. The ABTS^•+^ cation radical solution was prepared using ABTS solution (7 mM) and potassium persulfate (2.45 mM) in a ratio of 1:1, at 4 °C for 12–16 h, in the dark. A stock solution was then diluted with 80% ethanol solution, up to an absorbance of 0.700 ± 0.02, determined at 734 nm. Briefly, 10 μL of samples were mixed with 200 μL of ABTS^•+^ solution in a 96-well microplate. The plate was placed at room temperature for 30 min in the dark, and the absorbance was read at 734 nm. An SA standard curve was performed using a Trolox solution in ethanol (50 to 500 µM). ABTS values were expressed as micromoles of 6-hydroxy-2,5,7,8-tetramethylchroman-2-carboxylic acid (Trolox) equivalent (TE) per liter of *Aloe vera* juice (µmol TE/L).

#### 2.6.3. DPPH Radical Scavenging Activity

The DPPH radical scavenging activity was determined using the method described by Ferreira-Santos et al. [12], with minor modifications. The reaction was carried out in a 96-well microplate by mixing 25 µL of the sample and 200 µL of 2,2-diphenyl-1-picrylhydrazyl solution (DPPH, dissolved in 80% methanol to an absorbance of 0.700 ± 0.01 at 515 nm). Solutions were allowed to stand for 1 h in the dark, until complete reaction, at room temperature. Further, the absorbance was measured at 515 nm, using water as a blank. A standard curve was prepared with Trolox (40 to 400 µM). DDPH results were expressed as micromoles of Trolox equivalent (TE) per liter of *Aloe vera* juice (µmol TE/L).

### 2.7. Determination of Bioaccessibility

Bioaccessibility was determined using the method described by Simões et al. [26]. Briefly, 10 mL of digested *Aloe vera* juices (intestinal phase samples) were centrifuged at 18,000× *g* for 30 min, and two distinct phases were obtained: an opaque sediment phase at the bottom and a clear micelle phase at the top (supernatant). It was assumed that the model bioactive compounds were in the clear micelle phase (i.e., the bioaccessible fraction), which were available for absorption and metabolization. Then, the micelle phase was used to quantify the content of bioactive compounds released after in vitro digestion. Bioaccessibility was determined using the following equation:

(1) $$\mathrm{Bioaccessibility}\;\left(\%\right)=\frac{C_{\mathrm{Micelle}\;\mathrm{Phase}}}{C_{\mathrm{Intestinal}\;\mathrm{Phase}}}\times 100$$

where the C_Micelle phase_ is the concentration of bioactive compounds in the micelle phase, and the C_Intestinal phase_ is the concentration of bioactive compounds after in vitro digestion.

### 2.8. Statistical Analysis

Analyses were carried out in three independent replications of each sample. Results are represented as mean ± standard deviation (SD). Data were subjected to two-way ANOVA; pair-comparison of treatment means was obtained using the Bonferroni procedure at *p* < 0.05 and *p* < 0.001, using the statistical software GraphPad Version 6 (GraphPad Software Inc., San Diego, CA, USA).

## 3. Results

### 3.1. Effect of In Vitro Digestion on Phenolic Compounds

#### 3.1.1. Total Phenolic Content (TPC)

**Figure 2:**
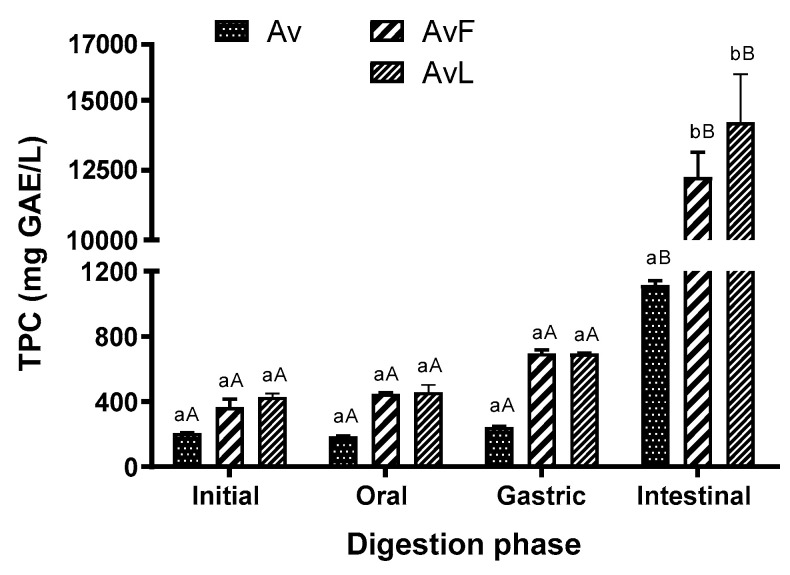
Total phenolic content of non-fermented and fermented *Aloe vera* juice at the digestion stages. *Av*: Digestion of non-fermented *Aloe vera* extract; *AvF*: Digestion of *Aloe vera* extract fermented with *E. faecium*; *AvL*: Digestion of *Aloe vera* extract fermented with *L. lactis*. Data bearing different lowercase letters (a, b) in the same digestion phase are significantly different (*p* < 0.05). Data bearing different capital letters (A, B) in the same sample groups are significantly different (*p* < 0.05).

The presence of some phenolic compounds in fruit and vegetable juices is of great importance since it determines their quality and influences their organoleptic characteristics, such as color and astringency, and their bioactive properties. Thus, in this study, the phenolic content of non-fermented and fermented *Aloe vera* juices was investigated during gastrointestinal digestion. In general, the behavior of phenolic compounds of fermented *Aloe vera* juices with *E. faecium* and *L. lactis* was more affected (positively) during the in vitro digestion than was the behavior of the non-fermented juice (Figure 2).

The Folin–Ciocâlteu method was used to determine the TPC, as it is commonly applied in food matrices and natural extracts [27,28]. The amount of TPC from non-fermented *Aloe vera* juice (*Av*), *Aloe vera* fermented with *E. faecium* (*AvF*), and *Aloe vera* juice fermented with *L. lactis* (*AvL*) was found to be significantly released during simulated in vitro digestion.

Before digestion, in the initial phase, the TPC were not significantly different between fermented and non-fermented juice (Figure 2), although after fermentation, the TPC became slightly higher in the fermented juices (207 ± 1 mg GAE/L for the Av, 366 ± 39 mg GAE/L for *AvF*, and 427 ± 17 mg GAE/L for *AvL* samples, respectively).

As expected, no significant differences between the initial and the oral phase were observed for all samples, since *Aloe vera* juice does not contain starch in its composition and therefore α-amylase was not mixed in the SSF.

In the gastric phase, the TPC for *Av* samples increased by 17% (up to 244 ± 4 mg GAE/L). Notably, for the fermented juices, TPC increased even more. For *AvF*, TPC increased up to 696 ± 16 mg GAE/L, and for *AvL* up to 695 ± 4 mg GAE/L, which was reflected in an increase of 90% and 62%, respectively, as compared to the amount determined in the initial samples. The low pH and the enzymatic action in the gastric digestion phase led to the release of some phenolic compounds bound with the carbohydrates, which increased the bioaccessibility of these bioactive compounds. The fermentation of the juice probably favored the positive results achieved in this study, since phenolic compounds increased in the gastric phase in comparison with other works where certain phenolic substances were lost in the stomach [29].

The great release of TPC was, however, determined in the last stage of digestion, at the intestinal phase. In this stage, an increase of 78% of TPC was observed for the non-fermented *Av* samples (1115 ± 23 mg GAE/L); the *AvF* fermented samples obtained an increase up to 12,253 ± 729 mg GAE/L, and the *AvL* up to 14,220 ± 1402 mg GAE/L, which was about a 95% increase as compared to the respective initial samples. In both the *AvF* and *AvL* samples, the amounts of TPC released during simulated intestinal digestion were significantly higher than in the earlier stages of digestion and in the undigested fermented juice (*p* value < 0.05).

#### 3.1.2. Phenolic Compounds of Non-Fermented *Aloe vera* Juice (*Av*)

**Figure 3:**
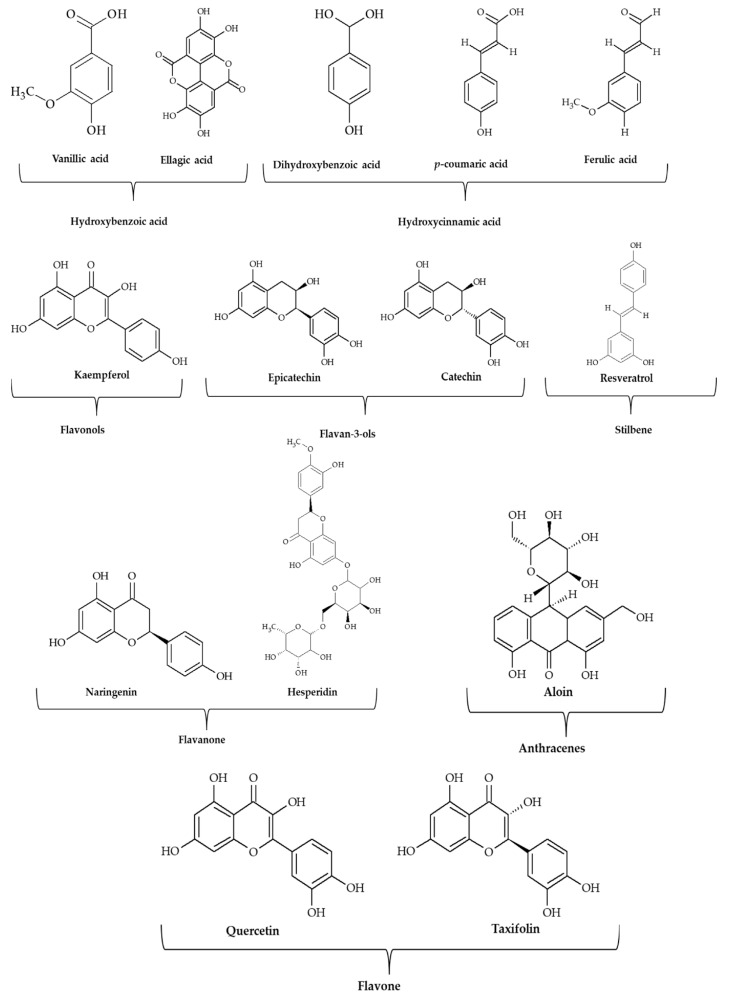
Bioactive compounds identified in *Aloe vera* juice.

Fourteen individual phenolic compounds were identified in the *Aloe vera* juice, whose structures are shown in Figure 3. Those phenolic compounds may be classified as hydroxybenzoic acids (vanillic, ellagic, and 3,4-hydroxybenzoic acids), hydroxycinnamic acids (*p*-coumaric and ferulic acids), flavonols (kaempferol), flavan-3-ols (epicatechin and catechin), stilbene (resveratrol), flavanones (naringenin and hesperidin), flavones (quercetin and taxifolin), and anthracenes (aloin).

All fourteen phenolic compounds selected for the UHPLC analysis were identified in the *Av*, as shown in Table 2. Epicatechin was the compound with the highest concentration in the initial juice (30.99 ± 2.95 mg/L). Its amount was maintained during the oral and gastric phases and drastically decreased in the intestinal phase (about 45%). Hesperidin, the compound with the second-highest concentration (17.01 ± 2.46), followed the same behavior. Aloin, a compound particularly from *Aloe vera*, was the third most concentrated in the *Av*, and decreased about 73% in the intestinal phase (*p* < 0.05). Naringenin (10.49 ± 0.87 mg/L) began to be degraded or biotransformed after the oral phase.

Ellagic acid was one of the hydroxybenzoic acids whose amount significantly increased during digestion (from 8.20 ± 1.31 mg/L in the juice up to 14.81 ± 0.03 mg/L in the intestinal phase). The content of flavonoids quercetin and kaempferol also increased significantly during the digestion process. It is important to note that kaempferol is obtained from naringenin by a biosynthetic pathway. The naringenin is converted into dihydrokaempferol, and dihydrokaempferol is converted into kaempferol [30]. Therefore, the decrease in naringenin concentration and increase in kaempferol concentration observed in all *Aloe vera* juices agrees with the expected theory.

Resveratrol, naringenin, taxifolin, catechin, and vanillic, as well as 3,4-dihydroxybenzoic, ferulic, and *p*-coumaric acids, were probably degraded or biotransformed during the digestion process, since their concentrations decreased (or were not detected) at the end of the digestion.

#### 3.1.3. Phenolic Compounds of *Aloe vera* Juice Fermentation with *E. faecium* (*AvF*)

For *AvF*, thirteen phenolic compounds were identified in its initial composition (Table 3). Catechin, being already in a low amount in the *Av*, was not detected in the fermented juice. It was observed that epicatechin was the compound with the highest concentration in *AvF* (35.29 ± 1.18 mg/L); however, its amount decreased significantly after the intestinal phase, as observed also for *Av*. This trend is in line with the results reported by Marchese et al. [31], which reported an important decrease in flavan-3-ols compounds after the digestion of green and black tea.

In the *AvF*, the ellagic acid was the compound with the third-highest concentration in the initial phase (15.09 ± 0.23 mg/L), almost twice the amount as compared to *Av* (8.20 ± 1.31 mg/L). Its concentration gradually increased throughout digestion, reaching an amount of 62.00 ± 1.53 mg/L at the intestinal phase, where its absorption begins. This phenolic acid was metabolized with the help of digestive enzymes and pH changes through digestion. As compared to *Av*, the ellagic acid of *AvF* juice increased three times more after the digestion process. Resveratrol, kaempferol and taxifolin also significantly increased during the digestion (152%, 600% and 15%, respectively).

Aloin, the major anthraquinonic component of *Aloe vera*, and one of the most important secondary metabolites of the plant due to its numerous functions as anti-inflammatory, anticancer, antibacterial, laxative, and purgative properties [26,27], increased during fermentation with *E. faecium*. However, its content decreased in the intestinal phase by up to 71% (*p* < 0.05). This trend was similar to the digestion of *Av* (76% of reduction). 

The content of naringenin and ferulic acid decreased during digestion, and quercetin was totally biotransformed or degraded at the intestinal phase. Other compounds like 3,4-dihydroxybenzoic, vanillic, and *p*-coumaric acids were quantified in small amounts in the initial phase, becoming not detected after the digestion. They showed to be sensitive to the changes in pH, and/or to the presence of salts and enzymes existent in the gastrointestinal system. 

Overall, the total amount of the identified phenolic compounds in *AvF* increased during digestion up to 24.5%. A total amount of 102 ± 10 mg/L was quantified in the initial phase and 127 ± 4 mg/L in the intestinal phase (Table 3).

#### 3.1.4. Phenolic Compounds of *Aloe vera* Juice Fermentation with *L. lactis* (*AvL*)

Only twelve phenolic compounds were identified in the *Aloe vera* juice fermented with *L. lactis* (*AvL*) (Table 4). The vanillic acid and catechin that were present in *Av* were possibly degraded or biotransformed during the fermentation with *L. lactis*. Similar to *Av* and *AvF*, epicatechin was the compound with the highest concentration in the initial phase (48.18 ± 2.19 mg/L), although it decreased by 83% during the stages of digestion.

The fermentation process of the *AvL* was also able to significantly increase (*p* < 0.05) the content of aloin in the juice, as for the *AvF*. Although its content had dropped 19% at the oral phase, afterward it remained constant. At the end of digestion, an amount of 15.59 ± 0.69 g/L was still quantified in the *AvL*, which was similar to the initial amount present in the *Av* and the *AvF*. Nevertheless, for *Av* and *AvF*, a high biotransformation or degradation occurred during digestion, decreasing up to 76% of its initial amount. 

Ellagic acid also increased from 15.39 ± 0.89 to 61.31 ± 3.71, similar as occurred for *AvF*. The content of hesperidin, resveratrol, and kaempferol also increased after digestion by 91%, 101%, and 473%, respectively. These phenolics also increased during the digestion of the *AvF*. This positive behavior was not observed in the digestion of the *Av*. Therefore, fermentation of *Av* with the LAB may promote the production of these compounds during digestion. 

Quercetin increased during the gastric phase (*p* < 0.05), and then, at the intestinal phase, was not detected. Apparently, ferulic acid was maintained during the stages of digestion. *p*-Coumaric and 3,4-dihydroxybenzoic acid was only detected in trace amount. Naringenin and taxifolin were decreasing in each phase of digestion, as shown in Table 4. 

Overall, for *AvL*, the concentration of phenolic compounds determined by liquid chromatography supports the results obtained by TPC analysis, which showed an increase of phenolics content in the intestinal phase relatively to the previous phases of the digestion. 

### 3.2. Bioaccessibility of Biocompounds from Aloe vera Juice

The bioaccessibility of individual phenolic compounds of *Aloe vera* juices is shown in Table 2, Table 3 and Table 4. During the digestion of *Av*, the highest percentage of bioaccessibility was determined for kaempferol (430%), followed by ellagic acid (195%), quercetin (160%), and resveratrol (72%). Meanwhile, epicatechin, *p*-coumaric acid, naringenin, hesperidin, ferulic acid, taxifolin, and aloin were found in the range of 55–3%. Vanillic acid, catechin, and 3,4-dihydroxybenzoic acid are more unstable compounds and therefore were degraded or biotransformed between the oral and the gastric phases of digestion.

Regarding the digestion of *AvF*, the highest percentage of bioaccessibility was also determined for kaempferol (699%), followed by ellagic acid (411%), resveratrol (252%), taxifolin (137%), hesperidin (112%), and ferulic acid (83%). Epicatechin, *p*-coumaric acid, naringenin, and aloin were found in the range of 57–29%. It was clearly observed that fermentation enhanced the bioaccessibility of phenolic compounds. The bioaccessibility determined for *AvL* had a similar trend as for *AvF*. The highest percentage of bioaccessibility was also determined for kaempferol (575%), followed by ellagic acid (398%), resveratrol (201%), hesperidin (194%), ferulic acid (99%), and aloin (76%), although taxifolin, naringenin, and epicatechin were found in the range of 62–17%. The actions of both human digestive and gut microbial enzymes represent an essential mechanism to metabolize polyphenols from components of the food matrix, which allows for the accessibility of the compounds [32]. Vanillic acid, catechin, *p*-coumaric acid, quercetin, and 3,4-dihydroxybenzoic acid did not resist the low pH of the gastric phase or any of the above-mentioned hypotheses.

### 3.3. Antioxidant Activity

**Figure 4:**
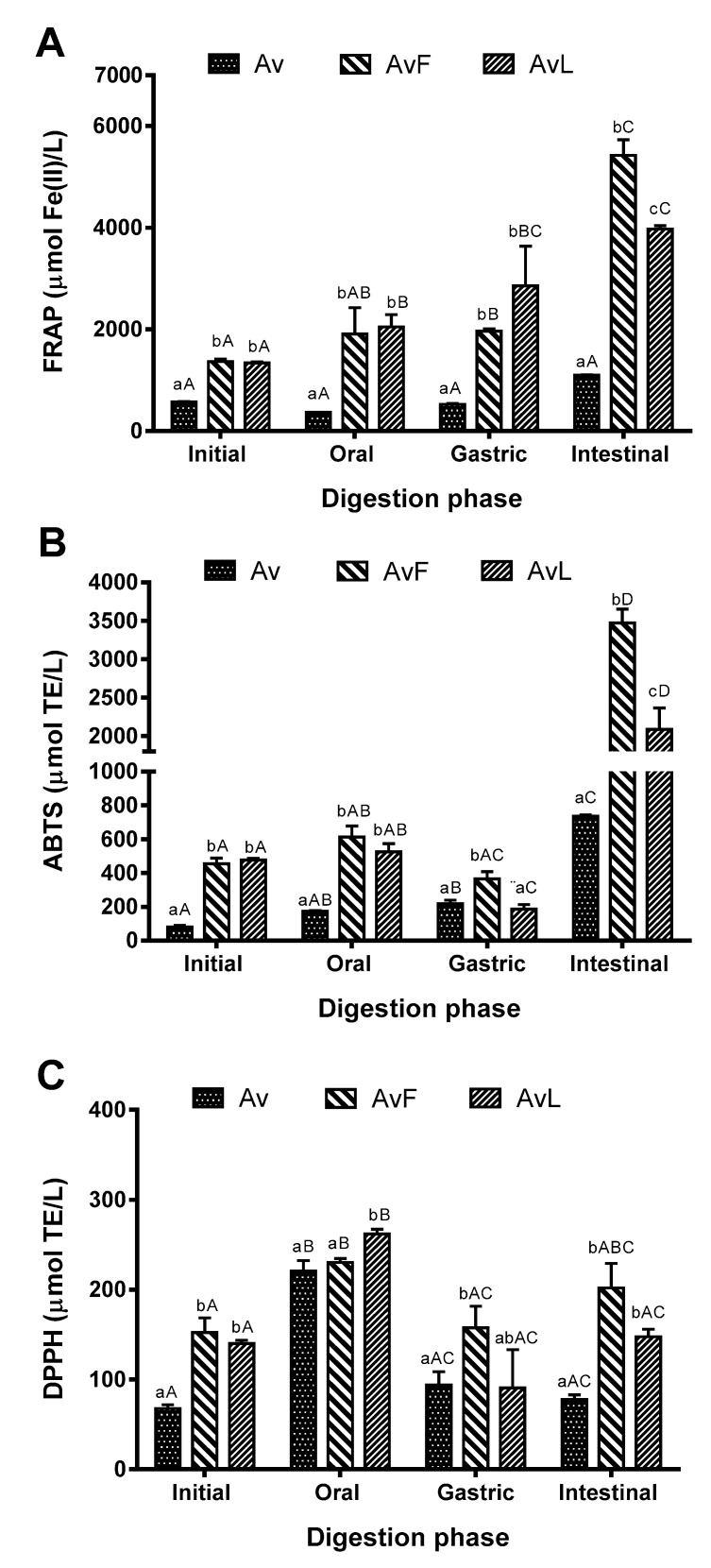
Antioxidant activity of non-digested and digested *Aloe vera* juices by (**A**) FRAP assay, (**B**) ABTS assay, and (**C**) DPPH assay. *Av*: Digestion of non-fermented *Aloe vera* extract; *AvF*: Digestion of *Aloe vera* extract fermented with *Enterococcus faecium*; *AvL*: Digestion of *Aloe vera* extract fermented with *Lactococcus lactis*. Data bearing different lowercase letters (a–c) in the same digestion phase are significantly different (*p* < 0.05). Data bearing different capital letters (A–D) in the same sample groups are significantly different (*p* < 0.05).

Three methods (FRAP, DPPH, and ABTS) were used to evaluate the antioxidant capacity of the undigested and digested *Aloe vera* juices (Figure 4), since each method provides an antioxidant capacity value related to its experimental conditions and reaction mechanisms [33]. The antioxidant activity of the *Aloe vera* juice significantly increased along the gastrointestinal digestion stages, according to the results obtained with the FRAP and ABTS assays. For the fermented juices (*AvF* and *AvL*), the determined antioxidant activity was even higher.

Results obtained by FRAP and ABTS for fermented samples (*AvF* and *AvL*) along the digestion steps were similar. Interestingly, a significant increase in antioxidant activity was observed for all juices during digestion, except for *Av*, whose antioxidant activity did not vary significantly during digestion when analyzed by FRAP. The most noticeable changes were observed for *AvF*.

On the other hand, the results of DPPH scavenging activity were opposite those of TPC (Figure 4C). It was observed that from the initial phase to the oral phase there was an increase in the antioxidant activity by DPPH. However, from the oral phase to the gastric phase, there was a decrease, and from the gastric phase to the intestinal phase, there was again an increase.

### 3.4. Correlation Analysis

As reported in the literature [34,35], the antioxidant activity of plant materials is mainly correlated with and affected by the phenolic content, as observed with the results obtained in this work. To evaluate the effect of TPC on the antioxidant activities of fermented and non-fermented *Aloe vera* digested samples, the correlations among them were analyzed by Pearson correlation. Results showed that there was a strong correlation among the antioxidant activity (FRAP and ABTS) of *Av* and of *AvL* with TPC (Table 5), suggesting that the antioxidant activity of the *Av* and *AvL* digested samples are dependent on their TPC. Therefore, phenolics released during digestion play an important role in the biological activity of the foods which, when absorbed, provide beneficial properties for consumers [35,36,37].

For *AvF* samples, however, the correlation among TPC and antioxidant activity (FRAP, ABTS, and DPPH) was insignificant. Nevertheless, the antioxidant activity of *AvF* increased after digestion. It is noteworthy that there was an increase in antioxidant activity, which could be associated with a specific phenolic compound such as epicatechin, which was found to be twice as high in the *AvF* juices as compared to the *AvL*.

The correlation analysis also showed that in the three *Aloe vera* samples there was no correlation among TPC and antioxidant activity using the DPPH method. This analysis suggests that the results of antioxidant activity by DPPH were independent of TPC, since one variable increases as the other decreases. This supports the results on the antioxidant activity obtained by DPPH assay of the three digested *Aloe vera* samples. The opposite behavior was observed in comparison with FRAP and ABTS, as the activity increased after digestion. This may be related with the differences in the methods applied for measuring antioxidant capacity.

## 4. Discussion

### 4.1. Effect of In Vitro Digestion on Phenolic Compounds

Polyphenols present in foods or beverages have been described as potential health promoters, although these benefits depend on their bioaccessibility in the gastrointestinal tract. To provide health benefits, compounds of interest must be released from the food matrix extraction and become bioaccessible in the gastrointestinal tract. These compounds must then be metabolized to reach the target tissue for action [38]. However, little is known about the stability during gastric and intestinal digestion and the metabolism of these phenolic compounds, suggesting that some phenolic fractions are absorbed and other fractions reach the colon, undergoing microbial transformations [39]. On the other hand, the intestinal microbiota are capable of metabolizing high molecular weight polyphenols into more biologically active metabolites. Thus, polyphenols support the prevention and treatment of several metabolic diseases, which are related to their antioxidant, anti-obesity, anti-inflammatory, anti-hypercholesterolemic, and antidiabetic properties [40].

In this sense, results gathered in this work suggest a transformation of high-molecular compounds or bound compounds into free compounds with lower molecular weight. These resulting polyphenol derivatives release reactive functional groups into a complex with the Folin–Ciocâlteu reagent, giving a visible increase in the TPC value. On the other hand, it has been reported that many phenolic compounds can be metabolized during digestion and absorbed in the intestine. This may be due to the fact that many polyphenols may not be free in the raw material and, after chemical extraction and further digestion, can be released into the small intestine, such as covalently bound or occluded [41]. Moreover, polyphenols can reduce the activity of digestion enzymes (e.g., pepsin, lipase); therefore, high concentrations of polyphenols may reduce the liberation of lipids and proteins, increasing the undigestible volume and, in turn, may result in increased amounts of polyphenols reaching the colon [42]. Qin et al. have also mentioned that phenolics and flavonoids are highly released during the intestinal phase [6], which is beneficial since phenolics are absorbed in the intestine. The results obtained in this study showed that the concentration of phenolics increased after gastric digestion, which promoted the bioaccessibility of the compounds, resulting in a healthier final product as a functional *Aloe vera* beverage.

Several studies showed that the release of phenolics begins in the oral or gastric phase [39,43,44]. The results of this work, however, showed that the highest release of phenolic compounds from probiotic *Aloe vera* juice takes place during or after the gastric phase. The differences in the amounts released of these active ingredients are related to differences in the compositions and bioactive properties among the different fruit/vegetable products [6]. In addition, during the gastrointestinal digestion of the *Aloe vera* juice, several changes could have occurred in the phenolic compounds such as (i) modification of the chemical structure, (ii) increase or decrease of solubility, or (iii) interaction with other compounds, which influenced their bioaccessibility [37].

However, these results were consistent with those reported by Qin et al. [6] since the highest release of health-related phenolic compounds from raspberry fruits and seeds was also observed during the intestinal phase. A similar association was demonstrated by Tarko and coworkers [45], who observed that the fermentation process did not significantly affect the amount of phenolic compounds contained in blackcurrant wines. Their study demonstrated that significant differences were only found after the action of digestive factors (enzymes, pH). The authors found that after each digestion stage of both blackcurrant must and wine, the total phenolic content increased [45].

Phenolics are considered the most important bioactive compounds, and their presence in foods is beneficial for consumers due to their health properties such as antioxidant, antimutagenic, and/or anticarcinogenic activities, anti-inflammatory action, and others [46]. In fact, phenolic compounds at low concentration may act as an antioxidant and protect foods and beverages from oxidative deterioration, which is of interest to the food industry [47].

### 4.2. Bioactive Compounds Identified in Aloe vera Juice

Overall, during the gastrointestinal digestion process of the non-fermented *Aloe vera* juice, the content of identified/quantified phenolic compounds decreased (Table 2). This decrease could be related to the instability of the phenols, which are then transformed into other molecules with higher or lower biological activity. This molecular transformation occurs at the OH groups attached to the benzene ring of the phenolic compounds, which structurally changes at high pH [48,49]. In this study, and for *Av*, the decrease in some bioactive compounds in the intestinal phase could be related to chemical reactions that promote the hydrolysis, oxidation, and polymerization of these compounds, resulting in the generation of other phenolic derivatives [49]. This is visible in the TPC result, which increases slightly throughout digestion (Figure 2).

After *Aloe vera* fermentation, it was possible to observe that some phenolic compounds increased. These compounds may be used or converted by LABs, *E. faecium* and *L. lactis*, during their metabolic activities. Meanwhile, ellagic acid, hesperidin, resveratrol, kaempferol, and taxifolin only increased during digestion, mainly in the fermented juices [50]. The increase in these compounds in the intestinal phase may be related to the elevated pH. Greater reactivity of phenolic compounds are observed at high pH values, which is assumed to be related to the formation of the phenolate ion, which promotes the reaction with peroxyl radicals as compared to that of the parent species [51].

No studies were found concerning the biotransformation of phenolic compounds from *Aloe vera* fermented with *E. faecium* and *L. lactis*. For this reason, the results obtained were compared with results obtained by other authors in other matrices. Previous studies run with cherry juice and broccoli puree fermented by different *Lactobacillus* strains also found a biotransformation of the phenolic compounds during fermentation [52]. These authors observed the synthesis of reduced compounds during food fermentation, demonstrating the biotransformation of some phenolic compounds (protocatechuic, caffeic and *p*-coumaric acids) in a synthetic substrate (MRS) using an LC–MS/MS apparatus [52].

As reported before, fermentation with LAB increased the ellagic acid content in *Av*. The behavior of ellagic acid during digestion was similar for *AvF* and *AvL.* The increase of this acid during fermentation may be related to the pH, with the presence of pancreatic enzymes, and with bile salts, factors that could promote the transformation of α- and β-punicalagins into ellagic acid. These results were consistent with results reported by Valero-Cases et al. [53] for fermented pomegranate juice, which had found an increase of ellagic acid in non-fermented pomegranate juice, but a decrease in fermented juice. The decrease was attributed to the generation of a new α-punicalagin derivative, which was only detected in the fermented juice, and was possibly a metabolite of the microbial transformations [53].

Ellagic acid and its derivatives are generated by hydrolysis of ellagitannins with acids or bases, when ester bonds are hydrolyzed, and the hexahydroxydiphenoyl group spontaneously rearranges into ellagic acid. Numerous derivatives of ellagic acid exist in plants, formed through methylation, glycosylation, and methoxylation of its hydroxyl groups. Furthermore, during food processing, ellagitannins are changed to free ellagic acid and ellagic acid derivatives [54]. This phenolic acid is probably released with the help of the enzymes and pH changes present in digestion, which results in its increased amount. Its absorption begins in the intestinal phase. This is important because the ellagic acid has been reported as a potential bioactive agent, with properties such as antibacterial, antiviral, antifibrotic, antiatherogenic, antimutagenic, anticancer, and strong antioxidant activity [55,56], increasing the potential health benefit of consumers.

Epicatechin decreased during the stages of digestion. This fact may suggest that the bacterial metabolism of epicatechin occurs during all steps of digestion, predominantly during the intestinal phase. The results agree with the work of Valero-Cases et al. [53], who observed a greater decrease in phenolic compounds such as flavan-3-ols in the intestinal phase for pomegranate juices fermented with *Bifidobacterium*.

Aloin decreased in the three *Aloe vera* samples. The results obtained for *Av* and *AvF*, where aloin decreased up to 76% after digestion, agree with those reported by Shim and Kwon [57]. They used an in vitro digestion model coupled with Caco-2 cells to assess the digestive stability and absorption of aloin, *Aloe*-emodin, and aloenin A. Aloenin A and *Aloe*-emodin were stable and entirely recovered during simulated digestion, but 50% of aloin was lost. On the other hand, for *AvL*, aloin decreased in the oral phase but then its amount was stable. The differences in the reduction of aloin in the *Aloe vera* juice when fermented either by *E. faecium* or *L. lactis* may be related to the metabolism of each bacterium. However, the reduction of aloin during the stages of digestion may be related to aloin-proteins binding and/or to its lower stability as compared to other bioactive components of *Aloe vera* [57]. Since aloin is metabolized by the gut microbiota into reactive *Aloe*-emodin, which is responsible for purgative activity and exhibits anti-cancer properties, its lower biodegradation in the upper gastrointestinal passage might be quite relevant [58].

### 4.3. Bioaccessibility of Biocompounds from Aloe vera Juice

The efficacy of functional foods in providing therapeutic or physiological benefits depends largely on the maintenance of their bioavailability, defined as the fraction of an ingested compound that is absorbed and available for physiological functions, i.e., that reaches the systemic circulation in an active form. Therefore, to exert their bioactivity, the active molecules have first to be bioaccessible, i.e., released from the food matrix and solubilized [59,60].

Contrary to numerous studies on medicinal properties of phenolic compounds in *Aloe vera*, there is limited information regarding the bioavailability and intestinal transport mechanism of these compounds. However, some studies simulate the digestion along the gastrointestinal tract, allowing the evaluation of their bioactivity. Existing static in vitro models simulate the digestion environment from the mouth to the intestinal phase. This model allows for the evaluation of changes in the bioaccessibility of phenolic compounds [59].

Results obtained in this work for *Aloe vera* showed that the bioaccessibility of phenolic compounds increased considerably, mainly in the samples that were fermented with *E. faecium* and *L. lactis*. Ellagic acid, hesperidin, resveratrol, ferulic acid, aloin, and kaempferol are compounds that were mainly favored with respect to bioaccessibility, which can be attributed mainly to the fermentation process of the samples. These results agree with the study by Campos-Vega et al. [61], which investigated the bioaccessibility and antioxidant capacity of spent coffee after gastrointestinal digestion and colonic fermentation. Their results concluded that digestion significantly increases the antioxidant activity, which is related to phenolic bioaccessibility.

The relationship observed in fermented juices between the phenolic compounds and microbial presence suggests the possible prebiotic effect of phenolic compounds on the LAB. The metabolites excreted by the *E. faecium* and *L. lactis* could produce health benefits due to the fact that fermentation enhances the bioavailability of *Aloe vera* juices.

### 4.4. Antioxidant Activity

Antioxidant compounds released from the food matrix, formed or transformed during the biological processes, or its synergistic activity, may have distinct behavior against different antioxidant assays, namely FRAP, ABTS, and DPPH.

The results obtained by ABTS are related to those obtained by FRAP and TPC analyses, since the intestinal phase is where there is the greater antioxidant activity after ingestion of *Aloe vera* juices, as observed in the intestinal phase with the methods used in this work. The greatest antioxidant activity determined by FRAP and ABTS in the intestinal phase was observed for *AvF*, which in turn improved the bioaccessibility of compounds. The increase in antioxidant activity after the intestinal phase could be explained by the additional time of reaction of the gastrointestinal simulation method (plus 2 h 45 min) and/or the effect of intestinal digestive enzymes (pancreatin and protease activity) on the complex food matrix, facilitating the release of compounds with antioxidant activity bound to the matrix [62]. In addition, the increase in antioxidant activity observed after gastrointestinal simulation suggests that the microorganisms involved in the fermentation process could act on the bioconversion of phenolic compounds, favoring antioxidant activity and bioaccessibility of these compounds [63].

Some authors have suggested that the probiotic fermentation process facilitates cleavage/dissociation of the bonds between phenolic compounds and other constituents, leading to the release of phenolic compounds-monomers, which increases antioxidant capacity [8,64].

The behavior for DPPH showed results contrary to those obtained by FRAP and ABTS, since in the oral and gastric phases, antioxidant activity increases, while in the intestinal phase, antioxidant activity decreases. This may be due to several factors, such as the molecular changes of some phenolics [53,65], the antioxidant activity testing methods employed, or with the interaction among the phenolics with the other constituents in the digestion samples [66]. It can possibly be explained by the influence of variations in the pH. The pH affects the racemization of molecules, leading to two chiral enantiomers with different bioavailability and, as a result, different bioactivity [65]. It has been reported that the influence of pH in polyphenols depends on the structure of the compound. Phenolic compounds with antioxidant capacity that are stable in acidic pH (gastric phase) do not necessarily maintain this capacity in neutral or alkaline pH (intestinal phase), which indicates that pH influences polyphenol stability as well as the possible occurrence of degradation or conversion of these compounds [67].

However, this contradiction among phenolic content and antioxidant activity was also found by other authors in other plant materials that were subjected to in vitro digestion. It has been reported in some works, such as a study by Ma et al. [35], that the antioxidant activity found using a DPPH test of digested pea shells was almost opposite that of TPC, which is in agreement with our results. In another study, the antioxidant activity of *Oxalis pes-caprae* extract was opposite the phenolic content [9]. Furthermore, the antioxidant activity increased after intestinal digestion.

The difference among the antioxidant capacity behaviors of the studied samples could be related to differences in the mechanisms accessed by these techniques. In the FRAP assay, antioxidant capacity is based on the antioxidant ability to reduce the ion iron III to iron II [33]. The ABTS method measures the antioxidant capacity to donate electrons and reduce the ABTS^•+^ radical [68]. The DPPH protocol is based on the antioxidant capacity of transferring hydrogen atoms to radicals [68]. Therefore, the phenolic compounds that are accessed in the FRAP method are not the same as those accessed in the ABTS or DPPH methods. In addition, the antioxidant activity of food products has been reported to increase, maintain, or decrease depending on the stability of the food components during gastrointestinal digestion and the properties of the derivatives obtained during the digestive process [32,49,69].

Therefore, antioxidants may play a role in the gastrointestinal tract by maintaining redox equilibrium against harmful oxidants, preventing gastrointestinal tract disorders and other diseases linked to reactive oxygen species (ROS) generation. The results obtained herein for *Aloe vera* demonstrated that the phenolics released during gastrointestinal digestion were able to reduce free radicals either by hydrogen donation (assessed by the ABTS test), or by electron donation (assessed by the FRAP test) (Figure 4A,B), possibly favoring hydrogen atom transfer mechanisms that require lower energy [66].

## 5. Conclusions

Overall, the present study demonstrated that fermentation of *Aloe vera* with *E. faecium* and *L. lactis* enhances the bioactivity of the *Aloe vera* active compounds, since the total phenolics increased more in the fermented *Aloe vera* than in the non-fermented juice during gastrointestinal digestion. The individual phenolic compounds, mainly ellagic acid, resveratrol, hesperidin, and kaempferol, increased after in vitro digestion and were more bioaccessible in the fermented *Aloe vera* juice. The antioxidant activities of the digested *Aloe vera* samples changed almost as a function of their TPC, and there were strong correlations among them (TPC and FRAP/ABTS) in *Aloe vera* fermented with *L. lactis*. This is reflected in the bioaccessibility, suggesting that *Aloe vera* fermented with LAB enhances health benefits, thereby justifying their valorization.

Further studies will, however, be necessary to understand the metabolization of the *Aloe vera* phenolic compounds by the gut microbiota and their impact and absorption by intestinal cells.

In conclusion, the fermentation of *Aloe vera* juice with *E. faecium* and *L. lactis* promoted the bioavailability of the compounds, transforming the non-fermented juice into a healthier beverage, with added nutrition and functionality.

## Figures and Tables

**Table 1:** Composition of electrolyte stock solutions: simulated salivary fluid (SSF), simulated gastric fluid (SGF), and simulated intestinal fluid (SIF).

	SSF (mmol/L)	SGF (mmol/L)	SIF (mmol/L)
KCl	15.10	6.90	6.80
KH_2_PO_4_	3.70	0.90	0.80
NaHCO_3_	13.60	25.00	85.00
MgCl_2_(H_2_O)_6_	0.15	0.12	0.33
(NH_4_)_2_CO_3_	0.06	0.50	-
NaCl	-	47.20	38.40
HCl	-	15.60	8.40

**Table 2:** Phenolic compound identification and quantification of undigested and in vitro digestion of non-fermented *Aloe vera* juice (*Av*). Data are given in mg/L.

Compounds (mg/L)	Initial Phase (Undigested)	Oral Phase	Gastric Phase	Intestinal Phase	Bioaccessibility (%)
Vanillic acid	1.87 ± 0.10 ^a^	1.30 ± 0.14 ^a^	n.d.	n.d.	n.d.
Catechin	1.19 ± 0.18 ^a^	n.d.	n.d.	n.d.	n.d.
Epicatechin	30.99 ± 2.95 ^a^	27.70 ± 1.38 ^a^	30.80 ± 4.24 ^a^	16.32 ± 0.41 ^b^	55.30 ± 1.2
*p*-Coumaric acid	3.30 ± 0.89 ^a^	3.61 ± 0.19 ^a^	2.41 ± 0.39 ^a^	0.09 ± 0.007 ^b^	2.62 ± 0.4
Ellagic acid	8.20 ± 1.31 ^a^	8.90 ± 0.68 ^a^	9.24 ± 0.37 ^a^	14.18 ± 0.03 ^b^	194.56 ± 0.2
Naringenin	10.49 ± 0.87 ^a^	9.95 ± 0.35 ^a^	6.46 ± 1.40 ^b^	2.27 ± 0.06 ^c^	22.01 ± 0.5
Hesperidin	17.01 ± 2.46 ^ab^	18.36 ± 0.85 ^a^	14.46 ± 1.77 ^b^	7.63 ± 0.32 ^c^	48.91 ± 0.9
Resveratrol	1.93 ± 0.07 ^a^	2.05 ± 0.07 ^a^	2.44 ± 0.14 ^a^	1.35 ± 0.01 ^a^	71.59 ± 0.0
Ferulic acid	4.43 ± 0.81 ^a^	3.59 ± 1.50 ^ab^	1.12 ± 0.50 ^b^	1.27 ± 0.01 ^b^	32.67 ± 0.3
Quercetin	0.75 ± 0.12 ^a^	4.27 ± 0.60 ^b^	3.01 ± 0.51 ^b^	1.24 ± 0.02 ^c^	160.45 ± 0.1
3,4-Dihydroxybenzoic acid	0.39 ± 0.18 ^a^	0.24 ± 0.02 ^a^	0.24 ± 0.04 ^a^	n.d.	n.d.
Taxifolin	7.53 ± 0.74 ^a^	6.88 ± 0.27 ^a^	6.69 ± 1.14 ^a^	1.78 ± 0.01 ^b^	25.12 ± 0.3
Aloin	14.10 ± 0.78 ^a^	13.47 ± 0.66 ^a^	14.86 ± 0.80 ^a^	3.40 ± 0.06 ^b^	25.03 ± 0.2
Kaempferol	1.53 ± 0.06 ^a^	1.40 ± 0.13 ^a^	1.36 ± 0.09 ^a^	6.42 ± 0.18 ^b^	430.24 ± 1.0
TOTAL	103.61 ± 11.51	101.71 ± 6.86	93.38 ± 11.38	55.95 ± 1.20	--

Values of phenolic compounds are expressed as concentration mean ± SD (mg/L) of three experiments. n.d.: not detected. Data bearing different lowercase letters (^a^–^c^) in the same compound are significantly different (*p* < 0.05) between different digestion phases.

**Table 3:** Phenolic compound identification and quantification of undigested and in vitro digestion of *Aloe vera* juice fermentation with *Enterococcus faecium* (*AvF*). Data are given in mg/L.

Compounds (mg/L)	Initial Phase (Undigested)	Oral Phase	Gastric Phase	Intestinal Phase	Bioaccessibility (%)
Vanillic acid	2.61 ± 0.02 ^a^	n.d.	n.d.	n.d.	n.d.
Epicatechin	35.29 ± 1.18 ^a^	22.97 ± 0.38 ^b^	7.61 ± 3.14 ^c^	17.52 ± 0.01 ^d^	49.7 ± 0.6
*p*-Coumaric acid	0.81 ± 0.02 ^a^	n.d.	n.d.	n.d.	n.d.
Ellagic acid	15.09 ± 0.23 ^a^	22.03 ± 0.48 ^b^	30.66 ± 0.43 ^c^	62.00 ± 1.53 ^d^	411.2 ± 0.9
Naringenin	4.44 ± 0.39 ^a^	6.0 ± 2.65 ^a^	3.52 ± 0.48 ^a^	2.51 ± 0.06 ^a^	57.1 ± 0.2
Hesperidin	10.03 ± 0.07 ^a^	13.10 ± 4.05 ^a^	14.35 ± 4.06 ^a^	11.25 ± 0.39 ^a^	112.2 ± 0.2
Resveratrol	3.27 ± 0.12 ^a^	4.13 ± 0.06 ^a^	5.34 ± 0.21 ^a^	8.23 ± 0.01 ^b^	251.7 ± 0.1
Ferulic Acid	6.01 ± 3.53 ^a^	3.42 ± 0.98 ^ab^	1.78 ± 0.23 ^b^	3.43 ± 0.24 ^ab^	83.3 ± 1.3
Quercetin	3.38 ± 1.96 ^a^	5.04 ± 0.22 ^a^	6.16 ± 0.47 ^a^	n.d.	n.d.
3,4-Dihydroxybenzoic acid	0.25 ± 0.01 ^a^	n.d.	n.d.	n.d.	n.d.
Taxifolin	4.25 ± 1.84 ^a^	5.18 ± 0.63 ^a^	3.06 ± 0.27 ^a^	4.88 ± 0.33 ^a^	137.2 ± 0.3
Aloin	15.49 ± 0.90 ^a^	13.61 ± 0.69 ^a^	13.84 ± 0.67 ^a^	4.46 ± 0.01 ^b^	28.9 ± 0.5
Kaempferol	1.88 ± 0.06 ^a^	2.73 ± 0.09 ^ab^	3.45 ± 0.04 ^b^	13.18 ± 2.32 ^c^	699.1 ± 1.1
TOTAL	102 ± 10	98 ± 10	89 ± 10	127 ± 4	--

Values of phenolic compounds are expressed as concentration mean ± SD (mg/L) of three experiments. n.d.: not detected. Data bearing different lowercase letters (^a^–^d^) in the same compound are significantly different (*p* < 0.05) between different digestion phases.

**Table 4:** Phenolic compounds identification and quantification of undigested and during in vitro digestion of *Aloe vera* juice fermentation with *Lactobacillus lactis* (*AvL*). Data given in mg/L.

Compounds (mg/L)	Initial Phase (Undigested)	Oral Phase	Gastric Phase	Intestinal Phase	Bioaccessibility (%)
Epicatechin	48.18 ± 2.19 ^a^	22.55 ± 3.72 ^b^	4.91 ± 0.72 ^c^	8.08 ± 2.86 ^c^	16.54 ± 1.4
*p*-Coumaric acid	0.38 ± 0.07 ^a^	n.d.	n.d.	n.d.	n.d.
Ellagic acid	15.39 ± 0.89 ^a^	21.97 ± 0.64 ^b^	30.30 ± 0.05 ^c^	61.31 ± 3.71^d^	398.21 ± 1.9
Naringenin	6.27 ± 0.04 ^a^	2.93 ± 1.06 ^b^	2.49 ± 1.13 ^b^	2.93 ± 0.21 ^b^	46.70 ± 0.1
Hesperidin	5.26 ± 0.72 ^a^	8.31 ± 1.76 ^ab^	5.28 ± 0.13 ^a^	10.03 ± 0.17 ^b^	193.77 ± 0.1
Resveratrol	4.01 ± 0.14 ^a^	4.44 ± 0.22 ^a^	5.14 ± 0.60 ^a^	8.07 ± 0.01 ^b^	201.40 ± 0.0
Ferulic acid	3.45 ± 0.07 ^a^	2.50 ± 0.28 ^a^	2.13 ± 0.44 ^a^	3.41 ± 0.13 ^a^	98.77 ± 0.0
Quercetin	1.13 ± 0.09 ^a^	3.07 ± 0.78 ^b^	4.96 ± 1.27 ^b^	n.d.	n.d.
3,4-Dihydroxybenzoic acid	0.24 ± 0.08 ^a^	n.d.	n.d.	n.d.	n.d.
Taxifolin	7.60 ± 0.60 ^a^	4.46 ± 0.74 ^b^	3.39 ± 0.22 ^b^	4.67 ± 0.0 ^b^	61.88 ± 0.0
Aloin	20.82 ± 2.33 ^a^	16.95 ± 2.11 ^ab^	16.89 ± 0.0 ^ab^	15.59 ± 0.69 ^b^	76.22 ± 0.3
Kaempferol	2.07 ± 0.10 ^a^	2.68 ± 0.17 ^a^	3.51 ± 0.0 ^a^	11.86 ± 0.31 ^b^	575.33 ± 0.2
TOTAL	114 ± 7	89 ± 11	78 ± 4	125 ± 8	--

Values of phenolic compounds are expressed as concentration mean ± SD (mg/L) of three experiments. n.d.: not detected. Data bearing different lowercase letters (^a^–^d^) in the same compound are significantly different (*p* < 0.05) between different digestion phases.

**Table 5:** Correlations between total phenolic content (TPC) and antioxidant activity (FRAP, ABTS, DPPH) of digested samples.

TPC	FRAP	ABTS	DPPH
Unfermented	0.9657 *	0.9843 *	−0.3807 ^ns^
Fermented with *E. faecium*	0.9246 ^ns^	0.9359 ^ns^	−0.0181 ^ns^
Fermented with *L. lactis*	0.9909 **	0.9934 **	0.2894 ^ns^

Significance of Pearson correlation coefficient (r) at *p* < 0.001 (**), *p* < 0.05 (*); no significance (^ns^).

## Data Availability

Not applicable.

## References

[1] Oliveira A., Amaro A.L., Pintado M. (2018). Impact of Food Matrix Components on Nutritional and Functional Properties of Fruit-Based Products. Curr. Opin. Food Sci..

[2] Mantzourani I., Terpou A., Bekatorou A., Mallouchos A., Alexopoulos A., Kimbaris A., Bezirtzoglou E., Koutinas A.A., Plessas S. (2020). Functional Pomegranate Beverage Production by Fermentation with a Novel Synbiotic L. Paracasei Biocatalyst. Food Chem..

[3] Valero-Cases E., Cerdá-Bernad D., Pastor J.J., Frutos M.J. (2020). Non-Dairy Fermented Beverages as Potential Carriers to Ensure Probiotics, Prebiotics, and Bioactive Compounds Arrival to the Gut and Their Health Benefits. Nutrients.

[4] Cuvas-Limon R.B., Nobre C., Cruz M., Rodriguez-Jasso R.M., Ruíz H.A., Loredo-Treviño A., Texeira J.A., Belmares R. (2020). Spontaneously Fermented Traditional Beverages as a Source of Bioactive Compounds: An Overview. Crit. Rev. Food Sci. Nutr..

[5] Cicenia A., Scirocco A., Carabotti M., Pallotta L., Marignani M., Severi C. (2014). Postbiotic Activities of Lactobacilli-Derived Factors. J. Clin. Gastroenterol..

[6] Qin Y., Wang L., Liu Y., Zhang Q., Li Y., Wu Z. (2018). Release of Phenolics Compounds from Rubus Idaeus L. Dried Fruits and Seeds during Simulated In vitro Digestion and Their Bio-Activities. J. Funct. Foods.

[7] Rasera G.B., de Camargo A.C., de Castro R.J.S. (2022). Bioaccessibility of Phenolic Compounds Using the Standardized INFOGEST Protocol: A Narrative Review. Compr. Rev. Food Sci. Food Saf..

[8] Morais S.G.G., da Silva Campelo Borges G., dos Santos Lima M., Martín-Belloso O., Magnani M. (2019). Effects of Probiotics on the Content and Bioaccessibility of Phenolic Compounds in Red Pitaya Pulp. Food Res. Int..

[9] Gonçalves S., Moreira E., Andrade P.B., Valentão P., Romano A. (2019). Effect of In vitro Gastrointestinal Digestion on the Total Phenolic Contents and Antioxidant Activity of Wild Mediterranean Edible Plant Extracts. Eur. Food Res. Technol..

[10] Žuntar I., Petric Z., Bursa’c D., Kovačevi’c B.K., Putnik P. (2020). Safety of Probiotics: Functional Fruit Beverages and Nutraceuticals. Foods.

[11] Bagci U., Ozmen Togay S., Temiz A., Ay M. (2019). Probiotic Characteristics of Bacteriocin-Producing Enterococcus Faecium Strains Isolated from Human Milk and Colostrum. Folia Microbiol..

[12] Choeisoongnern T., Sirilun S., Waditee-Sirisattha R., Pintha K., Peerajan S., Chaiyasut C. (2021). Potential Probiotic Enterococcus Faecium OV3-6 and Its Bioactive Peptide as Alternative Bio-Preservation. Foods.

[13] Huang J., Huang J., Yin T., Lv H., Zhang P., Li H. (2021). Enterococcus Faecium R0026 Combined with Bacillus Subtilis R0179 Prevent Obesity-Associated Hyperlipidemia and Modulate Gut Microbiota in C57BL/6 Mice. J. Microbiol. Biotechnol..

[14] Bandyopadhyay B., Das S., Kumar Mitra P., Kundu A., Mandal V., Adhikary R., Chandra Mandal N. (2022). Characterization of Two New Strains of Lactococcus Lactis for Their Probiotic Efficacy over Commercial Synbiotics Consortia. Brazilian J. Microbiol..

[15] Kondrotiene K., Lauciene L., Andruleviciute V., Kasetiene N., Serniene L., Sekmokiene D., Malakauskas M. (2020). Safety Assessment and Preliminary In vitro Evaluation of Probiotic Potential of Lactococcus Lactis Strains Naturally Present in Raw and Fermented Milk. Curr. Microbiol..

[16] Fernandes Pereira A.L., Rodrigues S. (2018). Turning Fruit Juice Into Probiotic Beverages. Fruit Juices: Extraction, Composition, Quality and Analysis.

[17] Cuvas-Limón R.B., Julio M.S., Carlos C.E.J., Mario C.H., Mussatto S.I., Ruth B.C. (2016). Aloe Vera and Probiotics: A New Alternative to Symbiotic Functional Foods. Annu. Res. Rev. Biol..

[18] Cuvas-Limón R.B., Ferreira-Santos P., Cruz M., Teixeira J.A., Belmares R., Nobre C. (2022). Novel Bio-Functional Aloe Vera Beverages Fermented by Probiotic Enterococcus Faecium and Lactobacillus Lactis. Molecules.

[19] Liu P., Chen D., Shi J. (2013). Chemical Constituents, Biological Activity and Agricultural Cultivation of Aloe Vera. Asian J. Chem..

[20] Baruah A., Bordoloi M., Deka Baruah H.P. (2016). Aloe Vera: A Multipurpose Industrial Crop. Ind. Crops Prod..

[21] Brodkorb A., Egger L., Alminger M., Alvito P., Assunção R., Ballance S., Bohn T., Bourlieu-Lacanal C., Boutrou R., Carrière F. (2019). INFOGEST Static In vitro Simulation of Gastrointestinal Food Digestion. Nat. Protoc..

[22] Nobre C., González A., Losoya C., Teixeira J.A., Belmares R., Abrunhosa L. (2022). Detoxification of Ochratoxin A and Zearalenone by Pleurotus Ostreatus during In vitro Gastrointestinal Digestion. Food Chem..

[23] González A., Nobre C., Simões L.S., Cruz M., Loredo A., Rodríguez-Jasso R.M., Contreras J., Texeira J., Belmares R. (2021). Evaluation of Functional and Nutritional Potential of a Protein Concentrate from Pleurotus Ostreatus Mushroom. Food Chem..

[24] Ferreira-Santos P., Genisheva Z., Pereira R.N., Teixeira J.A., Rocha C.M.R. (2019). Moderate Electric Fields as a Potential Tool for Sustainable Recovery of Phenolic Compounds from Pinus Pinaster Bark. ACS Sustain. Chem. Eng..

[25] Benzie I.F.F., Strain J.J. (1996). The Ferric Reducing Ability of Plasma (FRAP) as a Measure of “Antioxidant Power”: The FRAP Assay. Anal. Biochem..

[26] Simões L.S., Martins J.T., Pinheiro A.C., Vicente A.A., Ramos O.L. (2020). β-Lactoglobulin Micro- and Nanostructures as Bioactive Compounds Vehicle: In Vitro Studies. Food Res. Int..

[27] Mármol I., Quero J., Ibarz R., Ferreira-Santos P., Teixeira J.A., Rocha C.M.R., Pérez-Fernández M., García-Juiz S., Osada J., Martín-Belloso O. (2021). Valorization of Agro-Food by-Products and Their Potential Therapeutic Applications. Food Bioprod. Process..

[28] Ferreira-Santos P., Ibarz R., Fernandes J.-M., Pinheiro A.C., Botelho C., Rocha C.M.R., Teixeira J.A., Martín-Belloso O. (2021). Encapsulated Pine Bark Polyphenolic Extract during Gastrointestinal Digestion: Bioaccessibility, Bioactivity and Oxidative Stress Prevention. Foods.

[29] Xiang Z., Deng J., Yang K., Zhu Y., Xia C., Chen J., Liu T. (2021). Effect of Processing on the Release of Phenolic Compounds and Antioxidant Activity during In vitro Digestion of Hulless Barley. Arab. J. Chem..

[30] Duan L., Ding W., Liu X., Cheng X., Cai J., Hua E., Jiang H. (2017). Biosynthesis and Engineering of Kaempferol in Saccharomyces Cerevisiae. Microb. Cell Fact..

[31] Marchese A., Coppo E., Sobolev A.P., Rossi D., Mannina L., Daglia M. (2014). Influence of In vitro Simulated Gastroduodenal Digestion on the Antibacterial Activity, Metabolic Profiling and Polyphenols Content of Green Tea (*Camellia sinensis*). Food Res. Int..

[32] Castaldo L., Narváez A., Izzo L., Graziani G., Ritieni A. (2020). In Vitro Bioaccessibility and Antioxidant Activity of Coffee Silverskin Polyphenolic Extract and Characterization of Bioactive Compounds Using UHPLC-Q-Orbitrap HRMS. Molecules.

[33] Ferreira-Santos P., Genisheva Z., Botelho C., Rocha C., António Teixeira J. (2021). Valorization of Natural Antioxidants for Nutritional and Health Applications. Antioxidants—Benefits, Sources, Mechanisms of Action.

[34] Albishi T., John J.A., Al-Khalifa A.S., Shahidi F. (2013). Phenolic Content and Antioxidant Activities of Selected Potato Varieties and Their Processing By-Products. J. Funct. Foods.

[35] Ma Y., Gao J., Wei Z., Shahidi F. (2021). Effect of In vitro Digestion on Phenolics and Antioxidant Activity of Red and Yellow Colored Pea Hulls. Food Chem..

[36] Gullon B., Pintado M.E., Fernández-López J., Pérez-Álvarez J.A., Viuda-Martos M. (2015). In Vitro Gastrointestinal Digestion of Pomegranate Peel (*Punica granatum*) Flour Obtained from Co-Products: Changes in the Antioxidant Potential and Bioactive Compounds Stability. J. Funct. Foods.

[37] Lucas-Gonzalez R., Navarro-Coves S., Pérez-Álvarez J.A., Fernández-López J., Muñoz L.A., Viuda-Martos M. (2016). Assessment of Polyphenolic Profile Stability and Changes in the Antioxidant Potential of Maqui Berry (Aristotelia Chilensis (Molina) Stuntz) during In vitro Gastrointestinal Digestion. Ind. Crops Prod..

[38] Rein M.J., Renouf M., Cruz-Hernandez C., Actis-Goretta L., Thakkar S.K., da Silva Pinto M. (2013). Bioavailability of Bioactive Food Compounds: A Challenging Journey to Bioefficacy. Br. J. Clin. Pharmacol..

[39] Mosele J.I., Macià A., Romero M.P., Motilva M.J., Rubió L. (2015). Application of In vitro Gastrointestinal Digestion and Colonic Fermentation Models to Pomegranate Products (Juice, Pulp and Peel Extract) to Study the Stability and Catabolism of Phenolic Compounds. J. Funct. Foods.

[40] Fraga C.G., Croft K.D., Kennedy D.O., Tomás-Barberán F.A. (2019). The Effects of Polyphenols and Other Bioactives on Human Health. Food Funct..

[41] Bohn T., McDougall G.J., Alegría A., Alminger M., Arrigoni E., Aura A., Brito C., Cilla A., El S.N., Karakaya S. (2015). Mind the Gap—Deficits in Our Knowledge of Aspects Impacting the Bioavailability of Phytochemicals and Their Metabolites—A Position Paper Focusing on Carotenoids and Polyphenols. Mol. Nutr. Food Res..

[42] Bohn T. (2014). Dietary Factors Affecting Polyphenol Bioavailability. Nutr. Rev..

[43] Mosele J.I., Macià A., Romero M.P., Motilva M.J. (2016). Stability and Metabolism of Arbutus Unedo Bioactive Compounds (Phenolics and Antioxidants) under In vitro Digestion and Colonic Fermentation. Food Chem..

[44] Zheng G., Deng J., Wen L., You L., Zhao Z., Zhou L. (2018). Release of Phenolic Compounds and Antioxidant Capacity of Chinese Hawthorn “Crataegus Pinnatifida” during In vitro Digestion. J. Funct. Foods.

[45] Tarko T., Duda-Chodak A., Soszka A. (2020). Changes in Phenolic Compounds and Antioxidant Activity of Fruit Musts and Fruit Wines during Simulated Digestion. Molecules.

[46] Gullon B., Pintado M.E., Pérez-Álvarez J.A., Viuda-Martos M. (2016). Assessment of Polyphenolic Profile and Antibacterial Activity of Pomegranate Peel (Punica Granatum) Flour Obtained from Co-Product of Juice Extraction. Food Control.

[47] Karakaya S. (2004). Bioavailability of Phenolic Compounds. Crit. Rev. Food Sci. Nutr..

[48] Friedman M., Jürgens H.S. (2000). Effect of PH on the Stability of Plant Phenolic Compounds. J. Agric. Food Chem..

[49] Khochapong W., Ketnawa S., Ogawa Y., Punbusayakul N. (2021). Effect of In vitro Digestion on Bioactive Compounds, Antioxidant and Antimicrobial Activities of Coffee (*Coffea arabica* L.) Pulp Aqueous Extract. Food Chem..

[50] Yu Y., Zhang B., Xia Y., Li H., Shi X., Wang J., Deng Z. (2019). Bioaccessibility and Transformation Pathways of Phenolic Compounds in Processed Mulberry (*Morus alba* L.) Leaves after In vitro Gastrointestinal Digestion and Faecal Fermentation. J. Funct. Foods.

[51] Altunkaya A., Gökmen V., Skibsted L.H. (2016). PH Dependent Antioxidant Activity of Lettuce (L. Sativa) and Synergism with Added Phenolic Antioxidants. Food Chem..

[52] Filannino P., Bai Y., Di Cagno R., Gobbetti M., Gänzle M.G. (2015). Metabolism of Phenolic Compounds by *Lactobacillus* Spp. during Fermentation of Cherry Juice and Broccoli Puree. Food Microbiol..

[53] Valero-Cases E., Nuncio-Jáuregui N., Frutos M.J. (2017). Influence of Fermentation with Different Lactic Acid Bacteria and In vitro Digestion on the Biotransformation of Phenolic Compounds in Fermented Pomegranate Juices. J. Agric. Food Chem..

[54] Landete J.M. (2011). Ellagitannins, Ellagic Acid and Their Derived Metabolites: A Review about Source, Metabolism, Functions and Health. Food Res. Int..

[55] Kilic I., Yeşiloǧlu Y., Bayrak Y. (2014). Spectroscopic Studies on the Antioxidant Activity of Ellagic Acid. Spectrochim. Acta Part A Mol. Biomol. Spectrosc..

[56] Wang S.T., Chou C.T., Su N.W. (2017). A Food-Grade Self-Nanoemulsifying Delivery System for Enhancing Oral Bioavailability of Ellagic Acid. J. Funct. Foods.

[57] Shim S.M.I., Kwon H. (2010). Assessing Absorbability of Bioactive Components in Aloe Using In Vitro Digestion Model with Human Intestinal Cell. J. Food Biochem..

[58] Radha M.H., Laxmipriya N.P. (2015). Evaluation of Biological Properties and Clinical Effectiveness of Aloe Vera: A Systematic Review. J. Tradit. Complement. Med..

[59] Gonçalves R.F.S., Martins J.T., Duarte C.M.M., Vicente A.A., Pinheiro A.C. (2018). Advances in Nutraceutical Delivery Systems: From Formulation Design for Bioavailability Enhancement to Efficacy and Safety Evaluation. Trends Food Sci. Technol..

[60] Ting Y., Jiang Y., Ho C.T., Huang Q. (2014). Common Delivery Systems for Enhancing in Vivo Bioavailability and Biological Efficacy of Nutraceuticals. J. Funct. Foods.

[61] Campos-Vega R., Vázquez-Sánchez K., López-Barrera D., Loarca-Piña G., Mendoza-Díaz S., Oomah B.D. (2015). Simulated Gastrointestinal Digestion and In vitro Colonic Fermentation of Spent Coffee (*Coffea arabica* L.): Bioaccessibility and Intestinal Permeability. Food Res. Int..

[62] Barros R.G.C., Pereira U.C., Andrade J.K.S., de Oliveira C.S., Vasconcelos S.V., Narain N. (2020). In Vitro Gastrointestinal Digestion and Probiotics Fermentation Impact on Bioaccessbility of Phenolics Compounds and Antioxidant Capacity of Some Native and Exotic Fruit Residues with Potential Antidiabetic Effects. Food Res. Int..

[63] Liu Y., Cheng H., Liu H., Ma R., Ma J., Fang H. (2019). Fermentation by Multiple Bacterial Strains Improves the Production of Bioactive Compounds and Antioxidant Activity of Goji Juice. Molecules.

[64] Adebo O.A., Medina-Meza I.G. (2020). Impact of Fermentation on the Phenolic Compounds and Antioxidant Activity of Whole Cereal Grains: A Mini Review. Molecules.

[65] Wootton-Beard P.C., Moran A., Ryan L. (2011). Stability of the Total Antioxidant Capacity and Total Polyphenol Content of 23 Commercially Available Vegetable Juices before and after In vitro Digestion Measured by FRAP, DPPH, ABTS and Folin-Ciocalteu Methods. Food Res. Int..

[66] Bouayed J., Hoffmann L., Bohn T. (2011). Total Phenolics, Flavonoids, Anthocyanins and Antioxidant Activity Following Simulated Gastro-Intestinal Digestion and Dialysis of Apple Varieties: Bioaccessibility and Potential Uptake. Food Chem..

[67] Tagliazucchi D., Verzelloni E., Bertolini D., Conte A. (2010). In Vitro Bio-Accessibility and Antioxidant Activity of Grape Polyphenols. Food Chem..

[68] Schaich K.M., Tian X., Xie J. (2015). Hurdles and Pitfalls in Measuring Antioxidant Efficacy: A Critical Evaluation of ABTS, DPPH, and ORAC Assays. J. Funct. Foods.

[69] Vilas-Boas A.A., Oliveira A., Jesus D., Rodrigues C., Figueira C., Gomes A., Pintado M. (2020). Chlorogenic Acids Composition and the Impact of In vitro Gastrointestinal Digestion on Espresso Coffee from Single-Dose Capsule. Food Res. Int..

